# Novel amino acid metabolism‐related gene signature to predict prognosis in clear cell renal cell carcinoma

**DOI:** 10.3389/fgene.2022.982162

**Published:** 2022-09-02

**Authors:** Xiaofeng Cheng, Wen Deng, Zhicheng Zhang, Zhenhao Zeng, Yifu Liu, Xiaochen Zhou, Cheng Zhang, Gongxian Wang

**Affiliations:** ^1^ Department of Urology, The First Affiliated Hospital of Nanchang University, Nanchang, China; ^2^ Jiangxi Institute of Urology, Nanchang, China

**Keywords:** amino acid metabolism, clear cell renal cell carcinoma, gene signature, prognosis, machine learning

## Abstract

**Background:** Amino acid metabolism (AAM) deregulation, an emerging metabolic hallmark of malignancy, plays an essential role in tumour proliferation, invasion, and metastasis. However, the expression of AAM-related genes and their correlation with prognosis in clear cell renal cell carcinoma (ccRCC) remain elusive. This study aims to develop a novel consensus signature based on the AAM-related genes.

**Methods:** The RNA-seq expression data and clinical information for ccRCC were downloaded from the TCGA (KIRC as training dataset) and ArrayExpress (E-MTAB-1980 as validation dataset) databases. The AAM‐related differentially expressed genes were screened via the “*limma*” package in TCGA cohorts for further analysis. The machine learning algorithms (Lasso and stepwise Cox (direction = both)) were then utilised to establish a novel consensus signature in TCGA cohorts, which was validated by the E-MTAB-1980 cohorts. The optimal cutoff value determined by the “*survminer*” package was used to categorise patients into two risk categories. The Kaplan-Meier curve, the receiver operating characteristic (ROC) curve, and multivariate Cox regression were utilised to evaluate the prognostic value. The nomogram based on the gene signature was constructed, and its performance was analysed using ROC and calibration curves. Gene Set Enrichment Analysis (GSEA) and immune cell infiltration analysis were conducted on its potential mechanisms. The relationship between the gene signature and key immune checkpoint, N6-methyladenosine (m^6^A)-related genes, and sensitivity to chemotherapy was assessed.

**Results:** A novel consensus AMM‐related gene signature consisting of IYD, NNMT, ACADSB, GLDC, and PSAT1 is developed to predict prognosis in TCGA cohorts. Kaplan-Meier survival shows that overall survival in the high-risk group was more dismal than in the low-risk group in the TCGA cohort, validated by the E-MTAB-1980 cohort. Multivariate regression analysis also demonstrates that the gene signature is an independent predictor of ccRCC. Immune infiltration analysis highlighted that the high-risk group indicates an immunosuppressive microenvironment. It is also closely related to the level of key immune checkpoints, m^6^A modification, and sensitivity to chemotherapy drugs.

**Conclusion:** In this study, a novel consensus AAM-related gene signature is developed and validated as an independent predictor to robustly predict the overall survival from ccRCC, which would further improve the clinical outcomes.

## Introduction

Renal cell carcinoma has become one of the most prevalent genitourinary tumours (Siegel et al., 2021). Its incidence continues to increase, which accounts for approximately 5% of new cancer cases in males and 3% of female cases (Siegel et al., 2021). As the major subtype of renal cell carcinoma, clear cell renal cell carcinoma (ccRCC) makes up 70–80% of all cases with the highest mortality rate (Zhao et al., 2018). Meanwhile, only a subset of patients has yielded great benefit from targeted therapies due to the heterogeneity of ccRCC. The application of conventional classification was often not sufficient as it was determined only by several clinicopathological traits without regard to molecular biological features. Therefore, to tackle the abovementioned considerations and avoid latent overtreatment or undertreatment, it is imperative to identify reliable molecular signatures to optimise prognosis and predict immune responses in ccRCC.

Amino acid metabolism (AAM) deregulation, an emerging metabolic hallmark of malignancy, is geared toward the increased requirement of rapid cancer cell proliferation (Hanahan and Weinberg, 2011; Pavlova and Thompson, 2016). AAM plays an essential role in tumour proliferation, invasion, and metastasis (Vettore et al., 2020). As a super nutrient, glutamine was involved in a series of pathways in energy generation, macromolecular synthesis, and signalling transmission in cancer cells (Li and Zhang, 2016). Serine, glycine, and threonine and the one-carbon units these processes produce, such as methenyl and methyl, fulfil tumour cell growth and proliferation and maintain the cellular redox, genetic, and epigenetic status (Locasale, 2013; Liu et al., 2019). In addition, N6-methyladenosine (m^6^A) RNA methylation widely participate in the metabolic recombination of tumour cells (An and Duan, 2022). m^6^A, as one of the drivers of tumourigenesis and progression was closely associated with a variety of tumours (Yu et al., 2021). m^6^A RNA methylation also affects clinical prognosis by modulating the immune function of patients with ccRCC (Chen S. et al., 2020). Some studies have reviewed that individual AAM-related genes or gene signatures play a surprising role in tumour progression and have excellent prognosis prediction value in glioma and hepatocellular carcinomas (Seltzer et al., 2010; Liu et al., 2019; Zhao et al., 2021). Liu et al. (2019); Zhao et al. (2021) showed that the risk signature based on AAM-related gene could effectively predict prognosis in glioma and hepatocellular carcinomas, respectively. Previous studies showed that the AAM-related gene of large amino acid transporter-1 was closely associated with an unfavourable prognosis in ccRCC (Betsunoh et al., 2013). Likewise, logistic regression models constructed for several serum amino acids (histidine, glutamine, 1-methyl histidine, and norvaline) had superior predictive and prognostic values for ccRCC (Zhang et al., 2017). The tumor cells of ccRCC are highly dependent on glutamine as a result of the ubiquitous genetic loss of the von Hippel-Lindau tumour suppressor gene (Fu et al., 2019). Nevertheless, the characteristic of the AAM-related gene in ccRCC has not been comprehensively elucidated. Moreover, novel AMM-related gene signatures for predicting prognosis remain to be developed in ccRCC and their relationship with m^6^A needs to be further defined.

In this study, we endeavored to apply AAM-related genes to develop a novel consensus signature in the TCGA to assess the prognosis and the feature of the immune microenvironment, immune checkpoint, m^6^A modification, and chemotherapy response, which was validated in E-MTAB-1980 cohorts. This work may facilitate optimising precision treatments and improve the clinical prognosis of ccRCC patients.

## Material and methods

### Data collection

**FIGURE 1:**
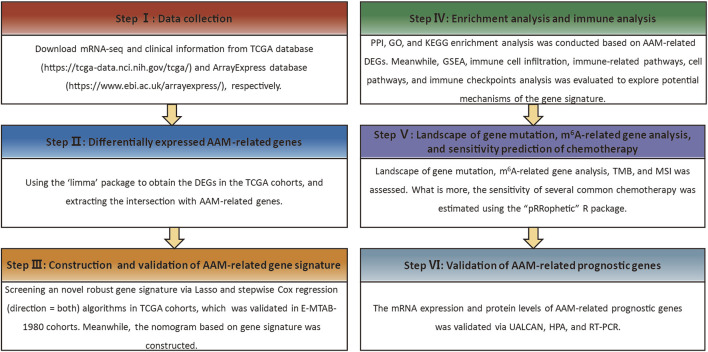
Workflow diagram.

The overall design of this study is illustrated in Figure 1. Five hundred and eighty ccRCC samples (including 508 tumours and 72 non-tumour tissues) were retrospectively downloaded, with the RNA-seq expression data (FPKM normalised data) and clinical information from kidney renal clear cell carcinoma (KIRC) of The Cancer Genome Atlas (TCGA) database (https://portal.gdc.cancer.gov/), which served as a training dataset. Data from the E-MTAB-1980 cohort was used as the validation dataset, and included RNA-seq transcriptomic data (normalised mRNA expression data) and clinical information of the complete 101 samples, obtained from the ArrayExpress database (https://www.ebi.ac.uk/arrayexpress/). The two datasets with complete overall survival (OS) information were used to construct and validate the stratification signature. The American Joint Committee on Cancer (AJCC) TNM staging system and the World Health Organization (WHO) grading classification were adopted in this study. The 374 AAM‐related genes were extracted from the gene sets (REACTOME METABOLISM OF AMINO ACIDS AND DERIVATIVES) obtained from the Molecular Signature Database v7.5 (MSigDB) (https://www.gsea-msigdb.org/gsea/msigdb/), as detailed in Supplementary Table S1.

The nine pairs of ccRCC tissues and adjacent tissue were retrieved from the First Affiliated Hospital of Nanchang University. The specimens were obtained with the patients’ informed consent and the approval of the Ethics Committee of the First Affiliated Hospital of Nanchang University. The study protocol was carried out following the guidelines of the Helsinki Declaration.

### Screen for AAM‐related differentially expressed genes

Differentially expressed genes (DEGs) between normal and ccRCC tissues were obtained with the criteria of adjusting *p* < 0.05 and | log2 fold change (FC) | > 1.5 via applying the “*limma*” package in the TCGA cohort. The intersecting AAM-related genes in the TCGA and E-MTAB-1980 cohorts were then identified. The “*VennDiagram*” package was applied to screen the co-expressed AAM-related DEGs.

### Functional enrichment analysis and protein-protein interaction network analysis

Gene Ontology (GO) and Kyoto Encyclopedia of Genes and Genomes (KEGG) pathway enrichment analyses were conducted using the ‘*clusterProfiler*’ package to explore the potential molecular mechanisms for AAM‐related DEGs (Yu et al., 2012). *p*-value < 0.05 and *q*-value < 0.05 were the criteria used to determine the significant enrichment function. Protein-protein interaction (PPI) analysis was performed via uploading the AAM‐related DEGs to the STRING database (http://www.string-db.org/), an online database of known and predicted protein-protein interactions (Szklarczyk et al., 2019). The results were then visualised using the Cytoscape software (version 3.7.1), where nodes denote proteins and edges represent interactions between these proteins. The ‘degree’ of a node was calculated by topological structure analysis of the PPI network.

### Construction and validation of the AAM-related prognostic signature

Univariate Cox regression based on AAM‐related DEGs is utilised to preliminarily screen interesting AMM-related genes heavily affecting the OS of ccRCC, to develop an AAM-related prognostic signature with high accuracy and stability performance in TCGA cohorts. The further analysis combined the least absolute shrinkage and selection operator (Lasso) and the stepwise Cox (direction = both) model was applied to determine the consensus risk stratification signature via the “*glmnet*” and “*survival*’” package, respectively. The risk score was calculated by following the formula: risk score = $\overset{n}{\underset{i=1}{\sum }}Coef_{i}\times \;Exp_{i}\;$ (where *Coef*_*i*_ was the coefficient of each gene weighted via multivariate Cox regression and *Exp*_*i*_ was the relative expression of each gene). All patients were divided into high- and low-risk groups according to the optimal cutoff value determined by the “*survminer*” package. The log-rank test and Kaplan-Meier curves were generated using the “*survival*” and “*survminer*” packages, respectively. To evaluate the predictive performance of the prognostic signature, the time-dependent receiver operating characteristic (ROC) curve was plotted using the “*timeROC*” package. Subsequently, the consensus risk stratification signature was validated using the E-MTAB-1980 cohorts via the abovementioned method.

### Correlation of the AAM-related gene signature with clinical traits

Multivariate Cox regression was conducted after adjusting for other available clinical traits to assess the predictive value of AMM-related gene signature. The nomogram with risk scores and commonly used clinical traits was constructed using the “*rms*” package to predict the OS of ccRCC. Harrell’s concordance index (C-index) was calculated to evaluate the accuracy of this model. The 1-year, 3-year, and 5-year calibration curves were also visualised to assess their predictive performance. Subsequently, the correlation between gene signature and clinical traits and subgroup analysis within ccRCC patients with various clinical traits was undertaken to further evaluate the predictive value of the risk stratification signature.

### Gene set enrichment analysis

Gene set enrichment analysis (GSEA) is a method of interpreting expression dataset using previously established gene sets defining pathways or functions. It aims to analyze the association between expression dataset and biological signals. The gene expression matrix and phenotype classes were uploaded to GSEA software V4.2 (https://www.gsea-msigdb.org/gsea/index.jsp) for GSEA with permutation = 100, min size = 15 and max size = 500 to further reveal potential mechanisms of prognostic signature.

### Immune cell infiltration, immune-related pathways, cell pathways, and immune checkpoints analysis

Given that the tumour immune microenvironment was essential for its prognosis in renal cell cancer (Giraldo et al., 2015), the Estimation of STromal and Immune cells in MAlignant Tumor tissues using Expression data (ESTIMATE) algorithm and cell-type identification by estimating relative subsets of RNA transcripts (CIBERSORT) algorithm was applied to evaluate the stromal score, immune score, and estimate score as well as 22 immune cell fractions content in patients between different groups, respectively. The results were then visualised via box and violin plots, respectively.

Additionally, a single-sample gene set enrichment analysis (ssGSEA) was performed using the R package ‘*GSVA*’ to assess differences in immune-related pathways and cell pathways. The reference genes on each immune-related pathway and cell pathway were reported in Supplementary Table S2 (Wei et al., 2020). The differences in the expression of key immune checkpoints between the two groups were compared as they are vital to tumour prognosis.

### Landscape of gene mutation and m^6^A-related genes analysis

The mutation frequencies and oncoplot waterfall plots for both groups of parients with ccRCC were generated by the “*maftools*” package.

Some studies consider that m^6^A is considered one of the drivers of tumorigenesis and progression in various tumours (Yu et al., 2021). Meanwhile, m^6^A RNA methylation widely participate in the metabolic recombination of tumour cells (An and Duan, 2022). m^6^A modification is closely related to the tumour immune landscape in ccRCC (Zhong et al., 2021). Thus, the expression level of the m^6^A-related gene between both risk groups was assessed to explore the potential mechanism of m^6^A modification in ccRCC. m^6^A-related genes were available from previously published articles, including regulators on writers, readers, and erasers (Sun et al., 2019; Zaccara et al., 2019).

### Tumour mutational burden, microsatellite instability, and sensitivity analysis of chemotherapy drugs

In tumour mutation burden (TMB) and microsatellite instability (MSI) analysis, the correlation between gene expression and TMB and MSI scores was evaluated by Spearman’s correlation analysis.

To explore the difference in several common chemotherapy sensitivity between both risk groups, the half-maximal inhibitory concentration (IC_50_) was estimated from the Genomics of Drug Sensitivity in Cancer database (https://www.cancerrxgene.org/) using the “*pRRophetic*” R package (Geeleher et al., 2014). The expression matrixes of both groups were uploaded to this database.

### Validation of AAM-related prognostic genes

The mRNA expression, promoter methylation, and protein expression levels of AAM-related prognostic genes in ccRCC were further obtained and validated from The University of ALabama at Birmingham CANcer data analysis Portal (UALCAN) database (http://ualcan.path.uab.edu/index.html) (Chandrashekar et al., 2017). Protein expression levels were also verified via immunohistochemistry (IHC) from the Human Protein Atlas (HPA) database (https://www.proteinatlas.org/).

The ccRCC and adjacent tissue were retrieved from ccRCC patients undergoing surgical treatment and were cryopreserved in liquid nitrogen after isolation. Total RNA was extracted from nine pairs of ccRCC tissue and adjacent tissue as well as renal normal or cancer cell lines HK-2, 786-O, A498, and OSRC-2 using Trizol reagent (ComWin Biotech, Beijing, China) and reversed transcribed into cDNA with the TransScript First-Strand cDNA Synthesis SuperMix kit (TransGen Biotech, Beijing, China) according to the manufacturer’s instructions. Real-time quantitative polymerase chain reaction (RT-PCR) was conducted with qPCR SYBR Green SuperMix (TransGen Biotech, Beijing, China). β-Actin was used, as an internal reference gene, to normalise the relative mRNA expressions with the 2^−ΔΔCT^ method. The primer sequences used in the study are listed in Supplementary Table S3.

### Statistical analysis

This study performed all statistical analyses and data processing using the R software (Version 4.1.3), with *p* < 0.05 considered statistically significant. The gene expression levels, risk scores, the abundance of immune cell infiltration, and drug’s IC_50_ between both risk groups were analysed via the Wilcoxon test, while immune scores (stromal score, immune score, and estimate score) and tumour purity via the unpaired Student’s t-test.

## Results

### Screen for AAM‐related DEGs and functional enrichment

**FIGURE 2:**
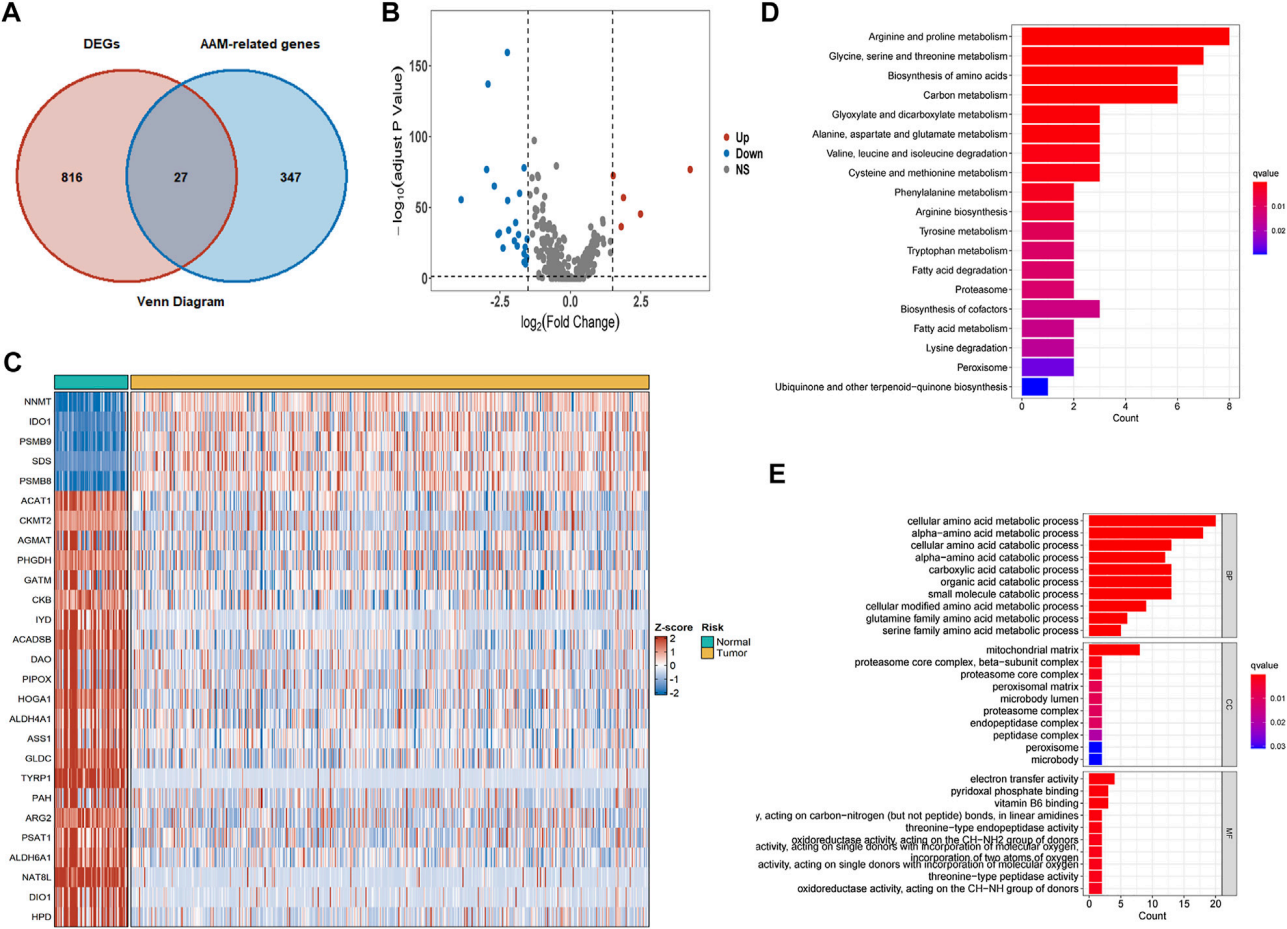
AAM‐related DEGs in TCGA-KIRC cohorts and functional enrichment analysis. **(A)** Venn plots of co-expression gene between DEGs and AAM-related gene **(B)** Volcano plots of AAM‐related DEGs; **(C)** Heatmap plots of AAM‐related DEGs; **(D)** KEGG analysis; **(E)** GO analysis involving biological process, cellular component, and molecular function.

Eight hundred forty-three DEGs were obtained from the intersection between differentially expressed genes in TCGA cohorts and genes in the E-MTAB-1980 cohorts. Subsequently, the AAM‐related DEGs were further elucidated (Figure 2A). Five of the 27 AAM‐related DEGs were highly expressed, while twenty-two were lowly expressed in tumour tissues for the TCGA cohort, as detailed in Figures 2B,C.

GO and KEGG analyses based on the 27 AAM‐related DEGs were then performed to assess the features of AAM-related genes and the biological processes involved (Figures 2D,E). KEGG analysis mainly was enriched in amino acid and carbon metabolism (Figure 2D). The GO analysis results indicated that biological processes (BP) were mainly enriched in cellular and alpha− amino acid metabolic and catabolic processes, cellular components (CC) in the mitochondrial matrix, as well as molecular functions (MF) in electron transfer activity (Figure 2E). Functional enrichment analysis corresponded to the molecular traits of AAM-related genes. The AAM-related DEGs might be involved in some biological processes such as AAM, electron transfer activity, fatty acid metabolism and degradation.

**FIGURE 3:**
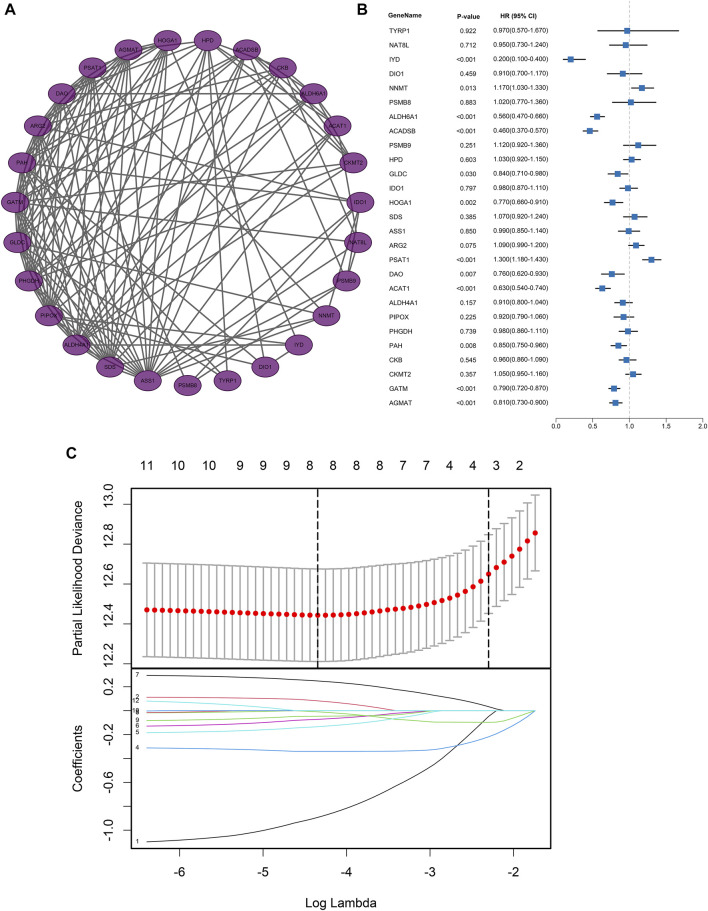
Protein-protein interaction network and regression analysis. **(A)** Protein-protein interaction network of AAM‐related DEGs; **(B)** Univariate Cox regression analysis of AAM‐related DEGs; **(C)** Lasso regression analysis of AAM‐related DEGs.

PPI analysis was also conducted to explore the relationship between the DEGs or corresponding proteins. The PPI network contained 27 nodes and 138 edges with a minimum required interaction score of low confidence (0.15), as depicted in Figure 3A.

### Development and validation of the AAM-related prognostic signature

Based on the expression profiles of AAM-related DEGs in the training dataset, univariate Cox regression regarding OS ultimately screened the 12 prognostic AAM-related genes, including Iodotyrosine Deiodinase (IYD), Nicotinamide N-Methyltransferase (NNMT), Aldehyde Dehydrogenase 6 Family Member A1 (ALDH6A1), Acyl-CoA Dehydrogenase Short/Branched Chain (ACADSB), Glycine Decarboxylase (GLDC), 4-Hydroxy-2-Oxoglutarate Aldolase 1 (HOGA1), Phosphoserine Aminotransferase 1 (PSAT1), D-Amino Acid Oxidase (DAO), Acetyl-CoA Acetyltransferase 1 (ACAT1), Phenylalanine Hydroxylase (PAH), Glycine Amidinotransferase (GATM), and Agmatinase (AGMAT) (Figure 3B). A combination model of Lasso and stepwise Cox (direction = both) was applied in TCGA cohorts to develop a consensus AAM-related gene signature, identifying a final set of 5 AAM-related DEGs. In this combination model, to avoid multicollinearity of several variables and overfitting the model, the Lasso regression regarding OS was employed to screen the key AAM-related DEGs. Based on the 10-fold cross-validation in the Lasso regression, the partial likelihood of deviance reached the minimum when the optimal lambda was 0.013. At that point, IYD, NNMT, ALDH6A1, ACDSB, GLDC, HOGA1, PSAT1, and ACAT1 were selected (Figure 3C). The stepwise Cox was utilised to further screen for genes associated with OS. Subsequently, each gene expression weighted by the regression coefficient in the multivariate Cox model based on the selected 5 AAM-related DEGs (AAM-related gene signature) was utilized to calculate the risk score with the formula as follows: Risk score = IYD × (-1.1404) + NNMT × (0.1243) + ACADSB × (-0.4140) + GLDC × (-0.2061) + PSAT1× (0.2789). All participants were assigned to high- or low-risk groups according to the optimal cut-off value.

**FIGURE 4:**
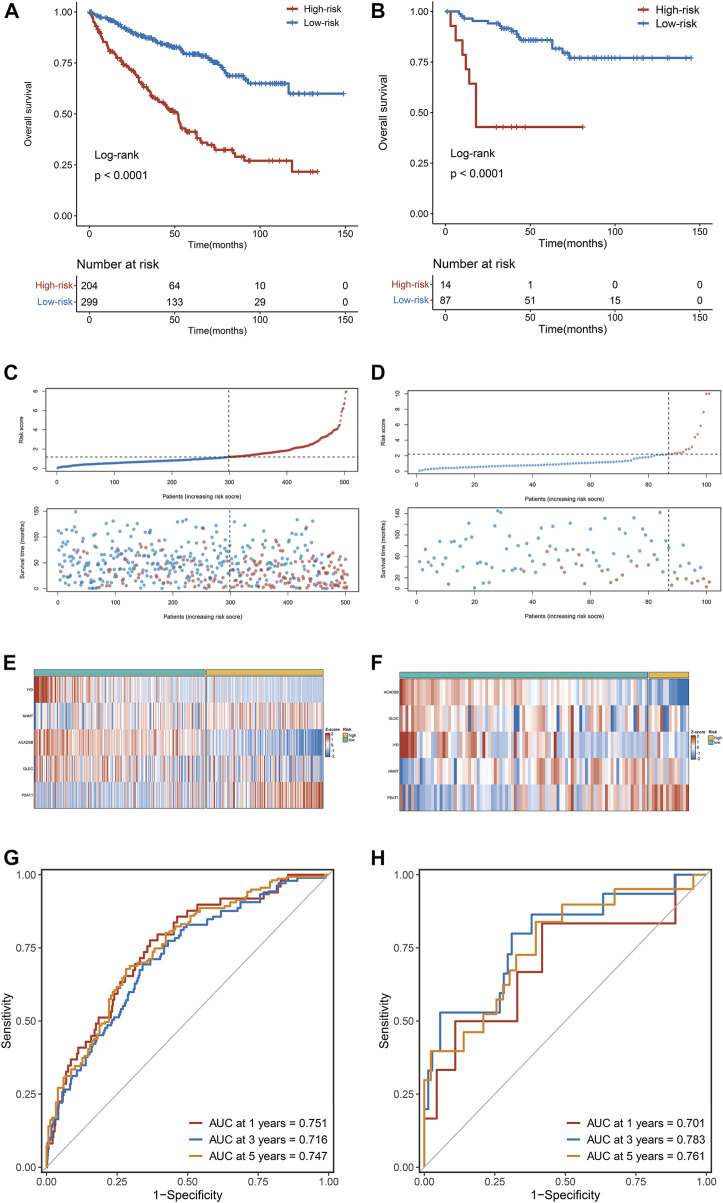
The correlation between the AAM-related gene signature and prognosis in ccRCC. **(A,B)** Kaplan-Meier survival analysis between high-risk groups and low-risk groups in TCGA **(A)** and E-MTAB-1980 **(B)** cohorts; **(C,D)** The trend in patient survival status as the increment of risk scores in TCGA **(C)** and E-MTAB-1980 **(D)** cohorts; **(E,F)** Heatmap plots of the prognostic genes in TCGA **(E)** and E-MTAB-1980 **(F)** cohorts; **(G,H)** Time-independent receiver operating characteristic (ROC) curve for predicting OS based on this signature in TCGA **(G)** and E-MTAB-1980 **(H)** cohorts.

Kaplan-Meier survival analysis showed that OS in the high-risk group was dramatically more dismal than in the low-risk group in the training dataset (TCGA cohort) (Figure 4A), validated only by the validation dataset (E-MTAB-1980 cohort) (Figure 4B). The risk score distribution and survival status for TCGA cohorts are depicted in Figure 4C and for E-MTAB-1980 cohorts in Figure 4D. The expression heatmaps of the five selected AAM-related genes for the TCGA cohorts and the E-MTAB-1980 cohorts are shown in Figures 4E,F, respectively. To evaluate the discrimination of the AAM-related prognostic signature, ROC analysis of OS is measured, and the 1-, 3-, and 5-year areas under the ROC curves (AUCs) were 0.751,0.716, and 0.747 in the TCGA cohorts (Figure 4G) and 0.701, 0.783, and 0.761 in the E-MTAB-1980 cohorts (Figure 4H), respectively.

### Establishment of a nomogram

**FIGURE 5:**
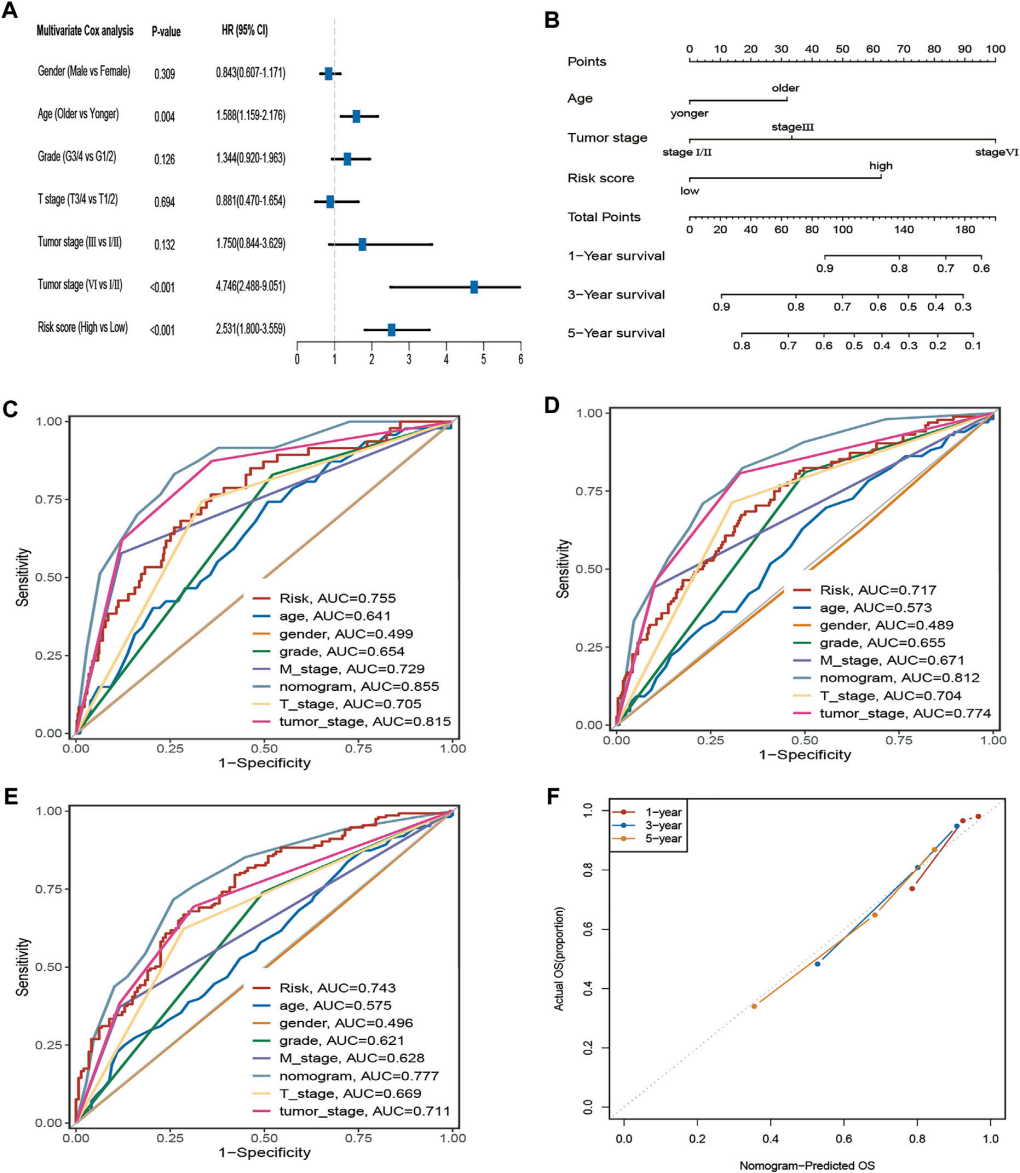
The AAM-related gene signature as an independent predictor for OS in TCGA cohorts and the predictive performance of the nomogram (risk models), risk (AAM-related gene signature), and other clinical traits. **(A)** The multivariate Cox regression analysis; **(B)** Nomogram for predicting 1-, 3-, and 5-year OS. **(C–E)** ROC curves for 1-year, 3-year, and 5-year OS based on the nomogram, risk, and other clinical traits, respectively; **(F)** the calibration plots for predicting 1-, 3-,5-year OS of the nomogram, respectively; T: AJCC TNM tumor stage; M: AJCC TNM node stage; G: WHO grade classification; tumor stage: AJCC tumor clinical stage.

Four hundred eighty-eight ccRCC patients in the TCGA cohort with complete clinical information were selected for further analysis. The multivariate Cox regression indicated that age, tumour stage, and risk score were independent prognostic factors (Figure 5A). Subsequently, the nomogram with age, tumour stage, and risk score was established to predict 1-, 3-, and 5-year OS in ccRCC patients from the TCGA cohort (Figure 5B). The prediction value of the nomogram is compared with the prognostic signature and other available clinical traits. As illustrated in Figures 5C–E, this nomogram model had greater AUCs at 1, 3, and 5 years. The C-index for the nomogram was 0.789 (95% confidence interval: 0.753–0.825). The 1-, 2-, and 3-year calibration curves for the nomogram showed that the actual OS was well in line with the predicted OS (Figure 5F).

### Relationship between AAM-related gene signature and clinical traits

**FIGURE 6:**
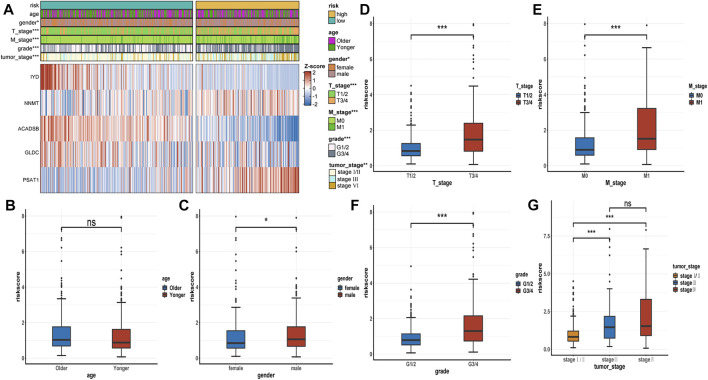
The correlation between the AAM-related gene signature and clinical traits. **(A)** Heatmap plot displayed the correlation between the risk group and other clinicopathological traits; **(B–G)** Boxplot showed the correlation between the risk scores and clinicopathological traits; T: AJCC TNM tumor stage; M: AJCC TNM metastasis stage; G: WHO grade classification; tumor stage: AJCC tumor clinical stage; ns: *p* ≥ 0.05; *: *p* < 0.05; **: *p* < 0.01; ***: *p* < 0.001.

**FIGURE 7:**
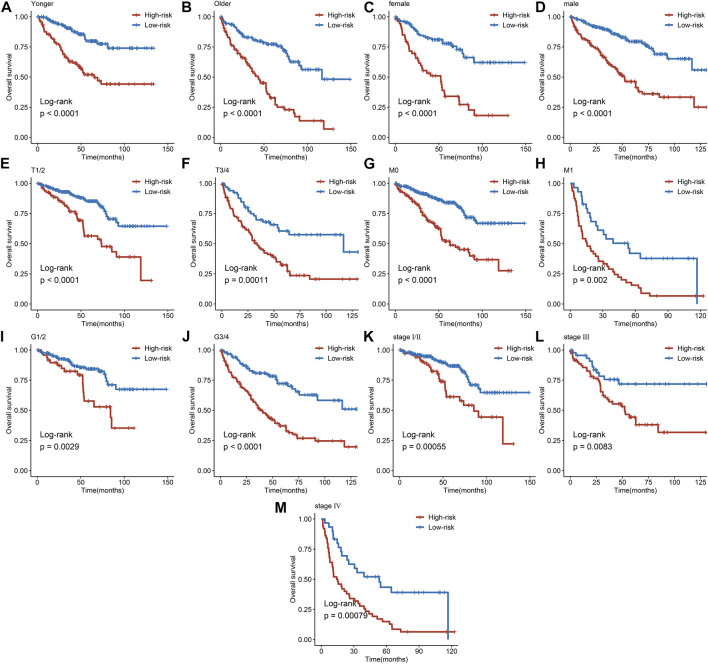
Subgroup analysis of the AAM-related prognostic gene signature according to age **(A, B)**, gender **(C, D)**, T stage **(E, F)**, M stage **(G, H)**, grade **(I, J)**, and tumor stage **(K, M)**; T, AJCC TNM tumor stage; M, AJCC TNM metastasis stage; G, WHO grade classification; tumor stage, AJCC tumor clinical stage.

The expression heatmap of the AAM-related prognostic signature indicated that the expression level of IYD, ACADSB, and GLDC in the high-risk group was lower than in the low-risk group. At the same time, NNMT and PSAT1 were highly expressed in the high-risk group, as shown in Figure 6A. It also demonstrated that the stratified risk was closely associated with the clinical metrics such as gender, T stage, M stage, grade, and tumour stage, consistent with the results in Figures 6B–G. The advanced clinical stage and grade have a higher risk score. To assess the prognostic predictive value of the AAM-related gene signature, the stratified subgroup analysis according to available clinical traits was conducted, suggesting that it encompasses reliable and accurate prediction ability (Figures 7A–M). The above results indicate that the novel gene signatures were closely related to several clinical traits, such as T stage, M stage, grade, and tumour stage.

### Gene set enrichment analysis

**FIGURE 8:**
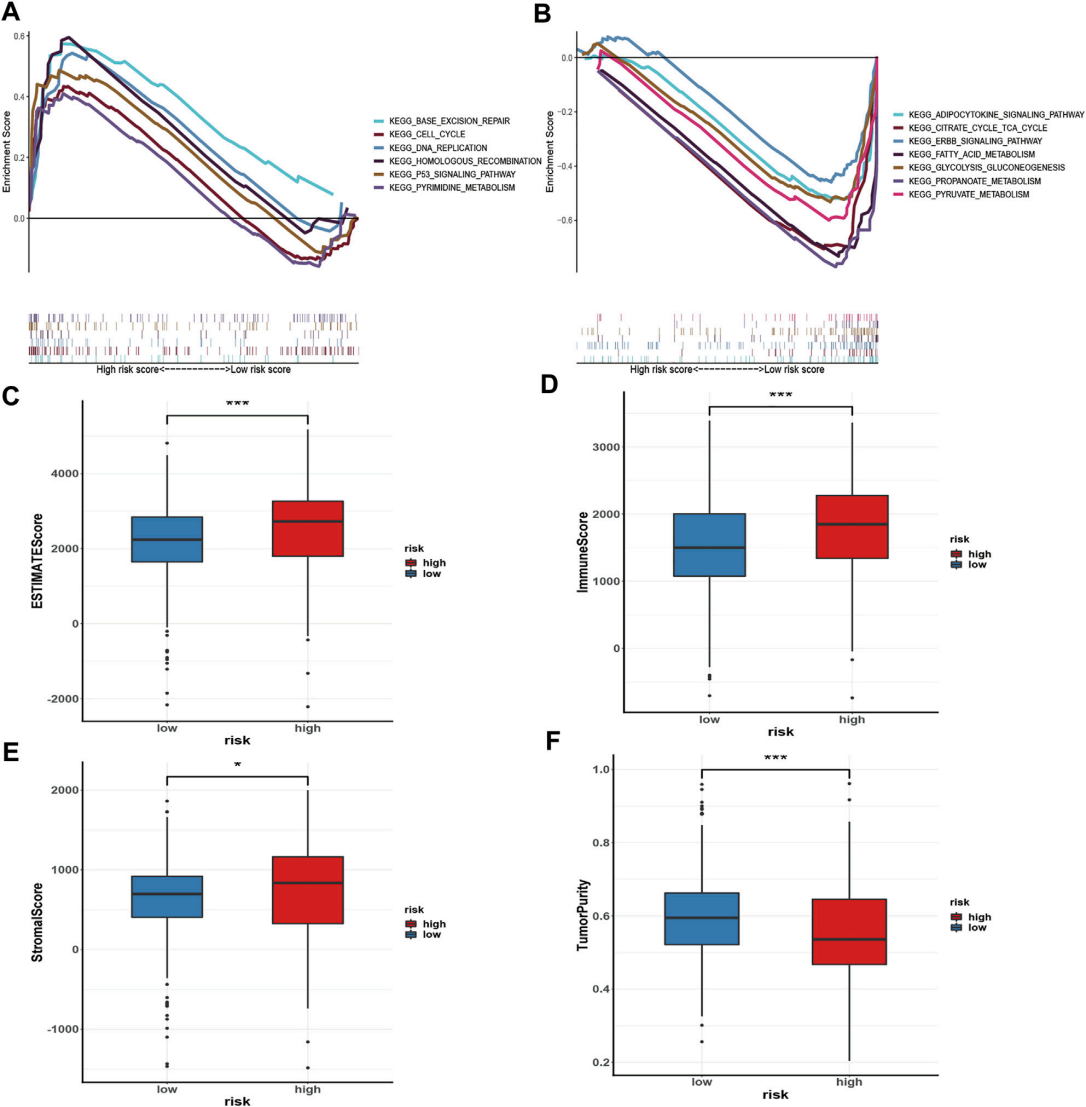
Gene set enrichment analysis in the high-risk group **(A)** and the low-risk group **(B)**, respectively; and Immune microenvironment analysis **(C–F)** *via* ESTIMATE algorithm, ns: *p* ≥ 0.05; *: *p* < 0.05; **: *p* < 0.01; ***: *p* < 0.001.

GSEA was performed to investigate the signalling pathways underlying the AMM-related risk signature, and demonstrates that the high-risk group was mainly enriched in base excision repair, homologous recombination, pyrimidine metabolism, the tumour protein p53 (p53) signalling pathway, DeoxyriboNucleic Acid (DNA) replication, and cell cycle (Figure 8A). Fatty acid metabolism, adipocytokine signalling pathway, citrate cycle, tricarboxylic acid cycle (TCA) cycle, propanoate metabolism, glycolysis, gluconeogenesis, pyruvate metabolism, and ERBB signalling pathway were primarily enriched in the low-risk group (Figure 8B).

### Immune cell infiltration, immune-related pathways, cell pathways, and immune checkpoint analysis

**FIGURE 9:**
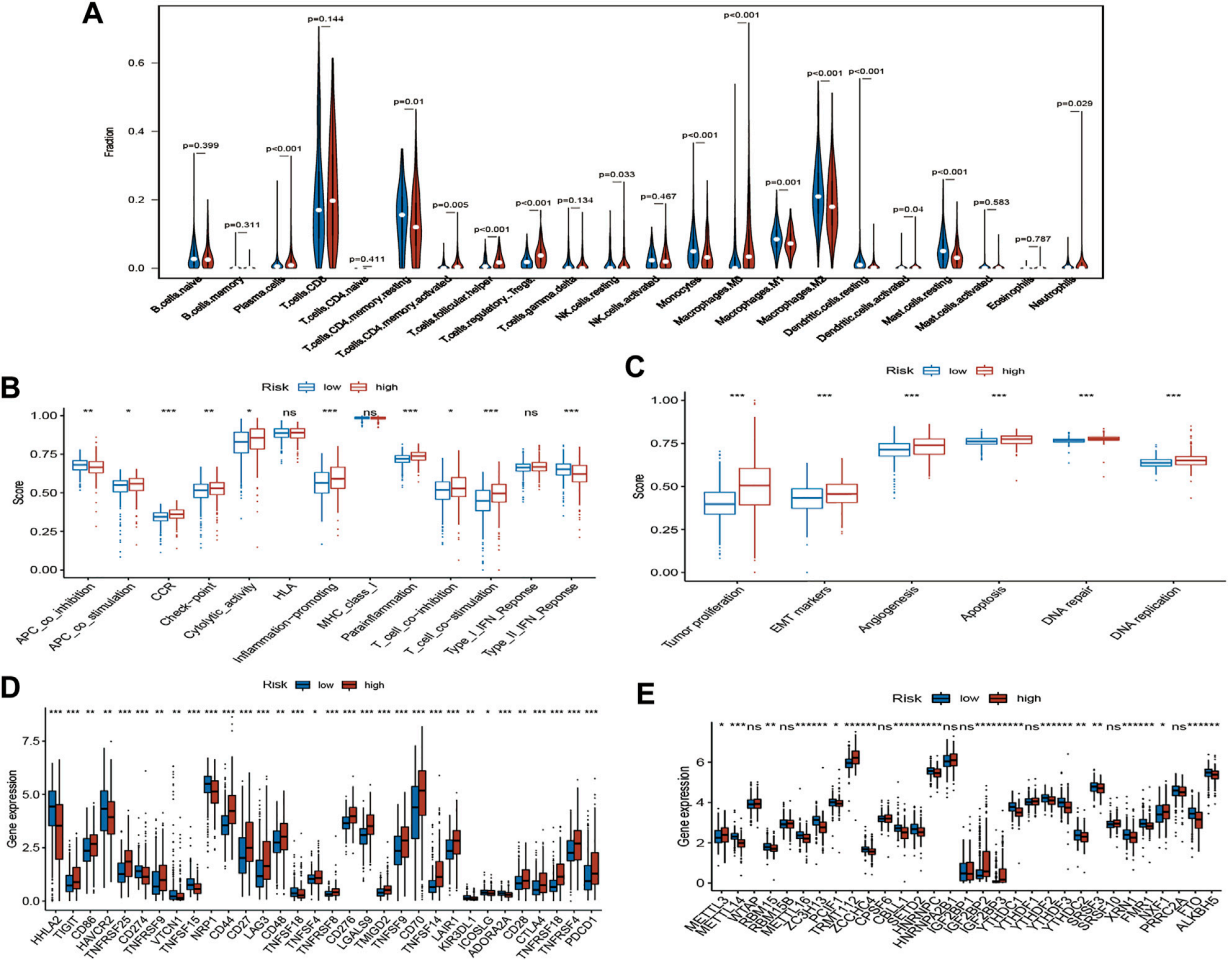
Immune infiltration, immune status and cell pathway evaluation, and immune checkpoints and m^6^A-related genes analysis. **(A)** The violin plot of immune cells proportion of the high-risk and low-risk group via CIBERSORT; The different immune status **(B)** and cell pathway **(C)** between the high-risk and low-risk group via ssGSEA; **(D)** Gene expression analysis of immune checkpoints between the high-risk and low-risk group; **(E)** Gene expression analysis of m^6^A-related genes between the high-risk and low-risk group; ns: *p* ≥ 0.05; *: *p* < 0.05; **: *p* < 0.01; ***: *p* < 0.001.

To evaluate the status of immune cell infiltration, immune-related pathways, and immune checkpoints, several appropriate algorithms such as ESTIMATE, CIBERSORT, and ssGSEA were employed. The estimate score, immune score, and stromal score in the high-risk group were significantly higher than in the low-risk group based on the ESTIMATE algorithm, in contrast to tumour purity (Figures 8C–F). The CIBERSORT results show that the proportion of plasma cells, resting and activated memory CD4 T cells, follicular helper T cells, regulatory Tregs T cells, resting NK cells, M0 macrophages, activated dendritic cells, and neutrophils in the high-risk group were elevated compared to the low-risk group. In contrast, the reverse was observed for monocytes, M1 and M2 macrophages, resting dendritic cells, and resting mast cells (Figure 9A). Furthermore, the ssGSEA demonstrated that most immune-related pathways such as antigen presenting cell (APC) co-stimulation, C-C chemokines receptors (CCR), checkpoint, cytolytic activity, inflammation-promoting, parainflammation, T cell co-inhibition, and T cell co-stimulation in the high-risk group had higher enrichment scores than in the low-risk group (Figure 9B). However, there was no difference between both risk groups in HLA, MHC class I, and type I IFN responses (Figure 9B). The enrichment score of cell pathways such as tumour proliferation, epithelial-mesenchymal transition (EMT), angiogenesis, apoptosis, DNA repair, and DNA replication were higher in the high-risk group (Figure 9C).

Notably, the expression of key immune checkpoints was also evaluated and demonstrated that most immune checkpoints such as PDCD1, CTLA-4, and CD28 are upregulated in the high-risk group. However, few immune checkpoints, such as PD-L1 (CD274) and HAVCR2, are downregulated (Figure 9D).

### Landscape of gene mutation and m^6^A-related genes analysis

The incidence of copy number variations and somatic mutations are summarized in Supplementary Figure S1; 254 of 330 (76.97%) ccRCC samples had genetic mutations. Missense mutation was the most common variant classification (Supplementary Figure S1A). Single nucleotide polymorphisms were the most common variant type, and C > T ranked as the top SNV class. The results also demonstrated VHL as the gene with the highest mutation frequency in both risk groups, followed by PBRM1 and TTN (Supplementary Figure S1B).

m^6^A RNA methylation widely participates in the metabolic recombination of tumour cells (An and Duan, 2022). The differential expression analysis of m6A-related genes was assessed because m^6^A is one of the drivers of tumorigenesis and progression in various tumours (Yu et al., 2021). For “Writers”, most genes, such as METTL14, RBM15, METTL16, ZC3H13, and PCIF1, were remarkably downregulated in the high-risk group, in contrast to METTL3. Similarly, the expression levels of various genes, such as ZCCHC4, YTHDC1, YTHDF2, YTHDF3, and YTHDC2, were significantly decreased in the high-risk group for “Readers”. FTO and ALKBH5 were also lowly expressed in the high-risk group with regard to “Erasers” (Figure 9E).

### TMB, MSI, and drug-sensitivity analysis

TMB and MSI can be used as a possible biomarker for predicting immunotherapy response in cancers (Chang et al., 2018; Samstein et al., 2019). To clarify the relationship between AAM-related gene signature and MSI as well as TMB, we then analysed the correlation between the prognostic gene and TMB as well as MSI. The results indicated a negative correlation between TMB and ACADSB (*p* = 0.003), but a positive correlation between TMB and NNMT (*p* = 0.004) as well as PSAT1 (*p* = 0.009). There was no correlation between TMB and IYD as well as GLDC (Supplementary Figure S2). IYD (*p* = 0.001) and NNMT (*p* = 0.048) were positively correlated with MSI. There was no significant correlation between MSI and other genes (Supplementary Figure S2).

**FIGURE 10:**
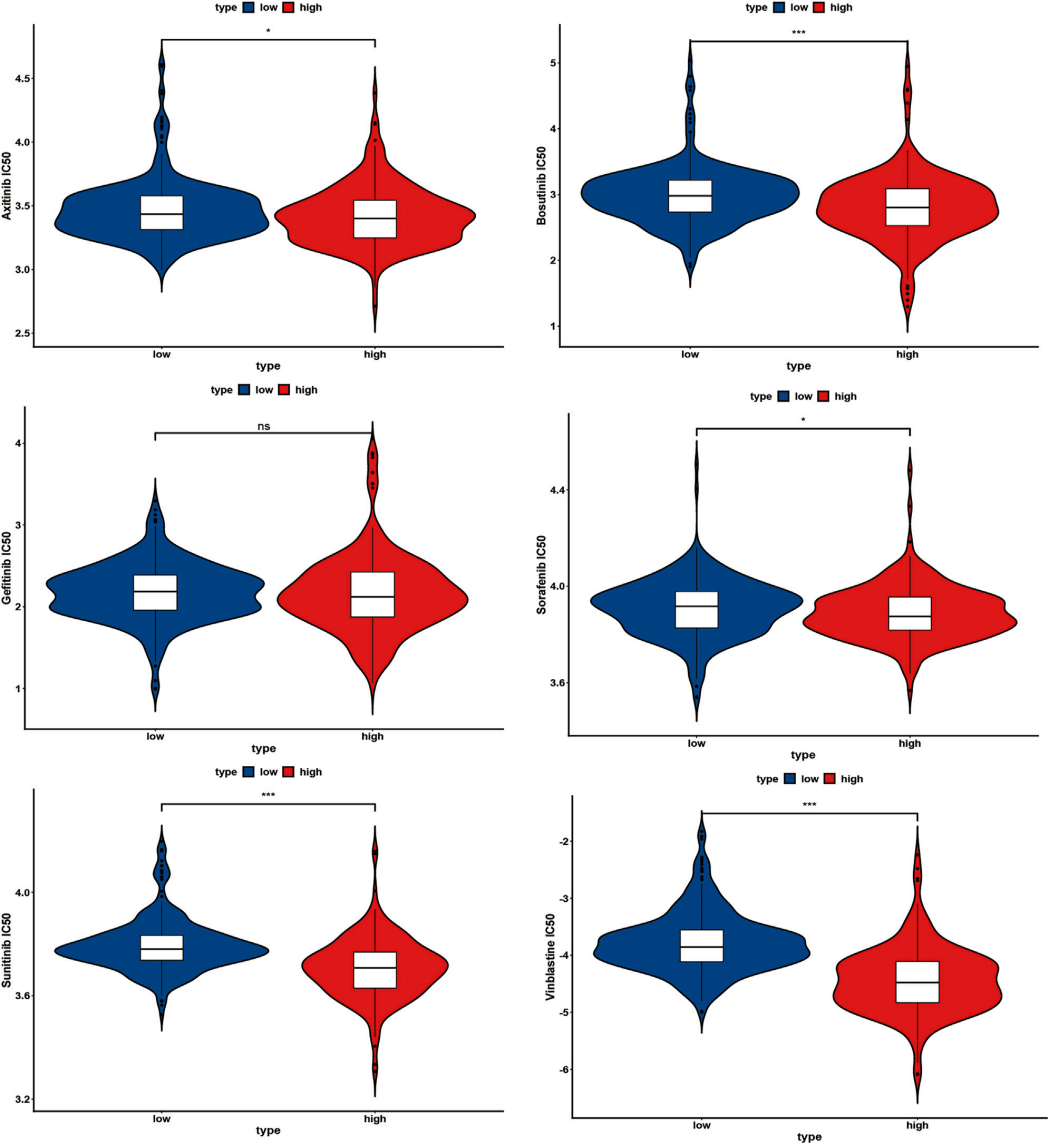
Several common chemotherapy drug sensitivities analyses between the high-risk and low-risk group, ns: *p* ≥ 0.05; *: *p* < 0.05; **: *p* < 0.01; ***: *p* < 0.001.

IC_50_ values for several chemotherapy drugs were measured as sensitivity metrics. The results suggest that participants in the low-risk group were more sensitive to several common chemotherapy drugs, including axitinib, bosutinib, sorafenib, sunitinib, and vinblastine, than in the high-risk group (Figure 10). However, there was no significant difference between both groups concerning the sensitivity of gefitinib (Figure 10).

### Validation of AAM-related prognostic genes

**FIGURE 11:**
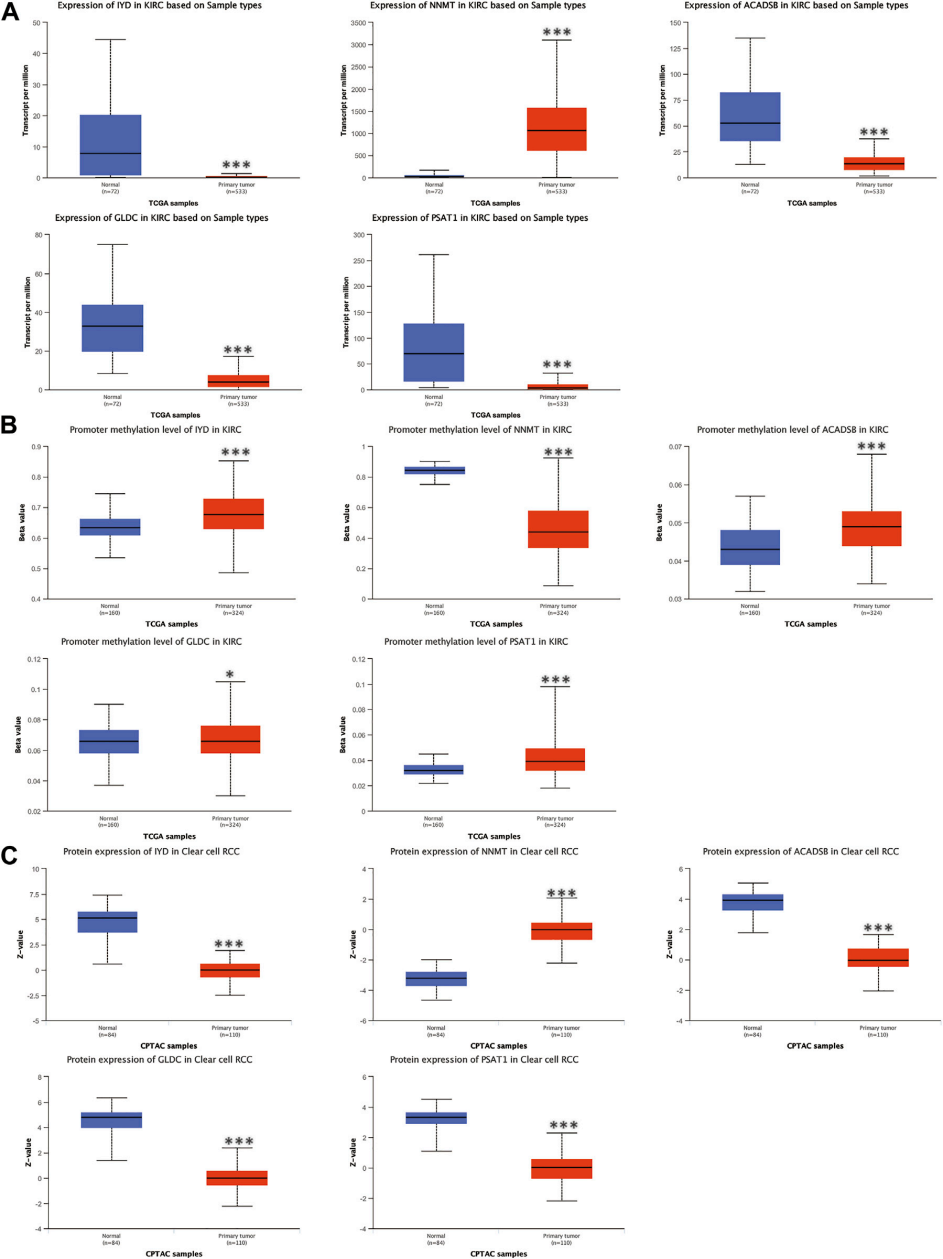
Validation of the mRNA expression levels **(A)**, promoter methylation levels **(B)**, and protein expression levels **(C)** of AAM-related prognostic genes via UALCAN database, ns: *p* ≥ 0.05; *: *p* < 0.05; **: *p* < 0.01; ***: *p* < 0.001.

**FIGURE 12:**
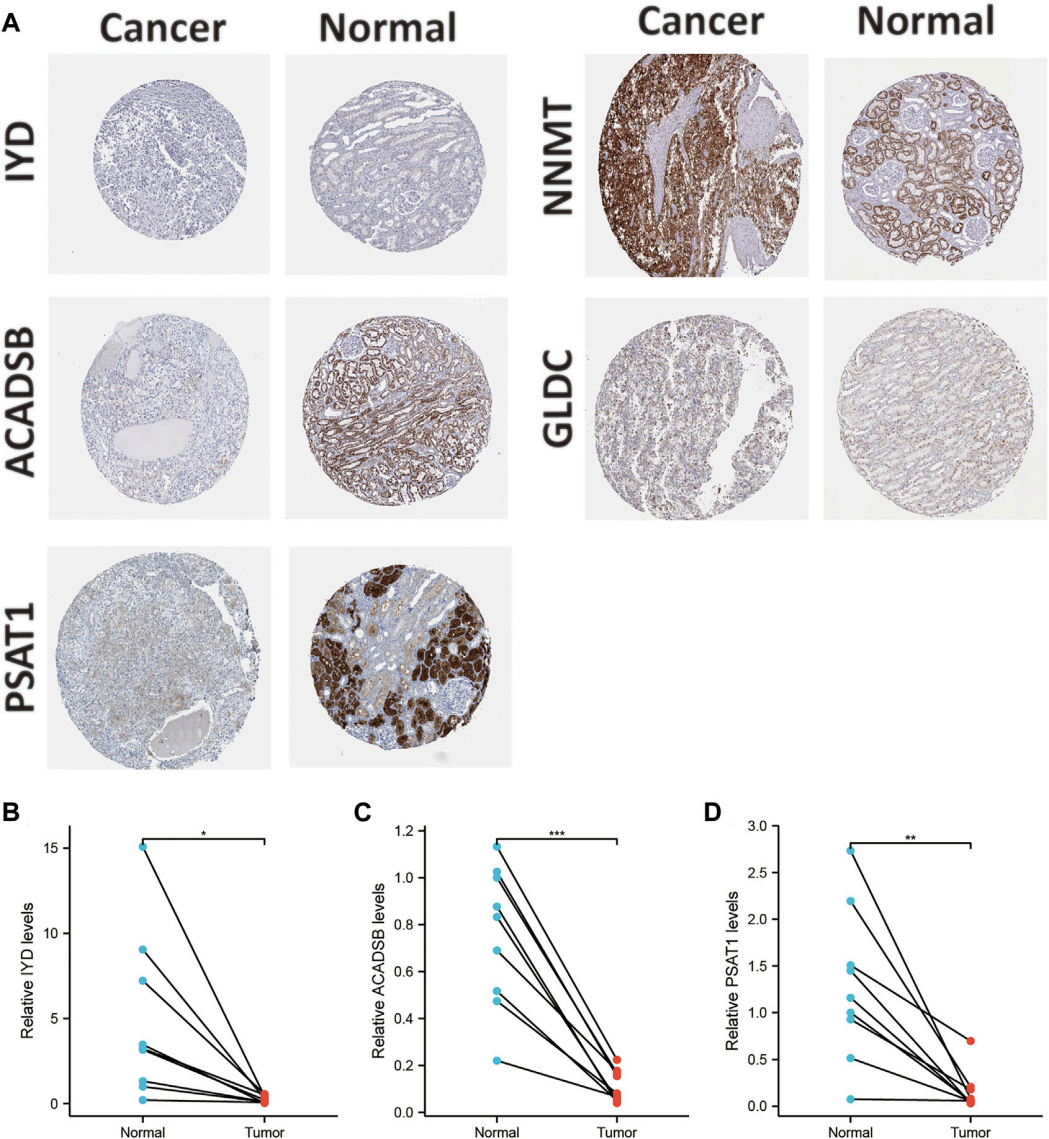
Validation of the protein expression levels of AAM-related prognostic genes via IHC in HPA database **(A)**; Validation of the gene expression levels of IYD **(B)**, ACADSB **(C)**, and PSAT1 **(D)** based on ccRCC and adjacent tissues via RT-PCR, ns: *p* ≥ 0.05; *: *p* < 0.05; **: *p* < 0.01; ***: *p* < 0.001.

The mRNA and protein expression levels of AAM-related prognostic genes were further validated via the UALCAN and HPA databases. The NNMT gene was highly expressed in primary tumour tissue compared with normal tissue. In contrast, several other genes (IYD, ACADSB, GLDC, and PSAT1) were under-expression (Figure 11A), which was in line with Figure 2C. Further, the promoter methylation level of prognostic genes showed the converse of the gene expression level above (Figure 11B). The protein expression level of several prognostic genes was also validated (Figures 11C, 12A), which was consistent with gene expression levels.

Additionally, Holstein et al. demonstrated that NNMT expression was apparently up-regulated in renal cancer tissues and cell lines (Holstein et al., 2019). NNMT also had a high protein expression in tissues and cell lines. Chen et al. also showed that GLDC expression was down-regulated in renal cancer tissues and cell lines (Chen Y. et al., 2020). The protein expression level of GLDC in tissues was in line with the RT-PCR results. Thus, this study selected IYD, ACADSB, and PSAT1 for validation since they had not been validated in renal cancer tissues and cell lines. The results of RT-PCR in tissues demonstrated that IYD, ACADSB, and PSAT1 had significantly lower expression levels in cancer tissues than in adjacent tissues, consistent with the predicted results (Figures 12B–D). However, only NNMT, GLDC, and ACADSB remained consistent with the abovementioned results in the renal cancer cell lines (Supplementary Figure S3).

## Discussion

The AJCC TNM staging system was implemented as a conventional clinical management tool to guide treatment decision-making. However, its limitations, such as heterogeneous clinical prognosis within the same stage patients, hamper the ability to provide optimal clinical care to patients as clinical traits rather than molecular features are primarily considered. Due to the pivotal importance of AAM for tumour proliferation, invasion, metastasis, and prognosis (Liu et al., 2019; Vettore et al., 2020; Zhao et al., 2021), the current study systematically established a novel consensus signature based on AAM-related genes to investigate the relationship between it and prognosis, immune infiltration, and sensitivity to various chemotherapeutic drugs.

With advancements in bioinformatic techniques, numerous predictive gene signatures have been proposed using various machine learning algorithms. This study develops a novel AMM-related gene signature according to the combination model of Lasso and stepwise Cox (direction = both). This algorithm can further reduce the dimension of variables to construct a robust gene signature more effectively, composed of 5 AAM-related genes. In addition, multivariate Cox regression, Kaplan-Meier survival, and ROC analyses suggest that this gene signature maintained high accuracy and reliability in the TCGA and E-MTAB-1980 cohorts, suggesting great potential for clinical risk stratification. This gene signature was closely associated with clinical traits (gender, T stage, M stage, grade, and tumour stage). To better understand the function of this gene signature in ccRCC, the individual AAM-related genes involved in this signature were highlighted. Previous studies demonstrated that the antioxidant-related gene signature constituted with IYD and five other genes could effectively forecast the prognosis of ccRCC and that IYD was closely associated with prognosis (Ren et al., 2021). Several studies revealed that NNMT was up-regulated, depending on the stage of progression in renal cell carcinoma, and its expression played a critical role in the invasive potential of human ccRCC cells (Tang et al., 2011; Holstein et al., 2019). Yue Wu et al. demonstrated that mitochondrial gene signatures, including ACADSB, could accurately predict OS, and ACADSB had good diagnostic and prognostic abilities in ccRCC (Liu et al., 2021; Wu et al., 2021). Yeda Chen et al. indicated that the expression levels of GLDC were significantly decreased in RCC cell lines compared to the normal cell lines (Chen Y. et al., 2020). Its expression level was down-regulated in RCC samples compared to those in paracancerous normal tissues. The function assay further showed that GLDC overexpression significantly inhibited the migration and invasion of RCC. Yan Zhang et al. reveal that the glycolysis-related gene signature based on PSAT1 had an excellent diagnosis and prognostic value in ccRCC (Zhang et al., 2021). The enrichment scores of cell pathways, such as tumour proliferation, epithelial-mesenchymal transition (EMT), angiogenesis, and apoptosis, were higher in the high-risk group, also suggesting that the AAM-related gene signature plays an essential role in the tumourigenesis and progression in ccRCC. Some studies demonstrated that tumours with higher levels of apoptosis are more aggressive (Morana et al., 2022). Thus, the above-selected gene, consisting of an AAM-related gene signature, plays an essential role in cancer progression and prognosis prediction for ccRCC.

This study also explored the gene signature’s latent biological mechanisms and immune infiltration features. GSEA indicated that cell growth and cell cycle pathways such as base excision repair were mostly enriched in the high-risk group. Likewise, the low-risk group’s lipid and glucose metabolism-related pathways were extraordinarily activated. The status of immune infiltration, immune-related pathways, and immune checkpoints were further evaluated using the appropriate algorithms. The estimate score, immune score, and stromal score are remarkably elevated in the high-risk group, which suggested elevated immune activity in this subgroup. When CIBERSORT was used to assess the proportions of 22 immune cell subsets, the results showed that most immune cells, including plasma cells, resting and activated memory CD4 T cells, follicular helper T cells, regulatory Tregs T cells, resting NK cells, M0 macrophages, activated dendritic cells, and neutrophils, were significantly higher in the high-risk group than in the low-risk group. Regulatory T cells (Tregs) are up-regulated in high-risk tumours. Tregs as immunosuppressive cells helps to suppress antitumour T cell responses. It can yield immunosuppressive cytokines and immune-inhibitory receptors that impair the activation of antitumour T cells (Speiser et al., 2016). Macrophages, found in all stages of tumour progression, were particularly abundant in the tumour. It was also immunosuppressive, preventing tumour cell attacks by natural killer and T cells (Noy and Pollard, 2014). M1 (antitumour) and M2 (pro-tumour) phenotypes may represent various function states rather than truly different cell types (Noy and Pollard, 2014). Zhang et al. (2019) demonstrated that the high proportion of M0 macrophages was closely associated with worse poor outcomes, which might reflect their gradual function differentiation. Immune functions are evaluated via the ssGSEA algorithm, which indicate that the APC co-stimulation, CCR, immune checkpoint, cytolytic activity, inflammation-promoting, parainflammation, T cell co-inhibition, and T cell co-stimulation were up-regulated in the high-risk group. Appropriate stimulation of APCs is essential for initiating and maintaining the immune response (Gallucci and Matzinger, 2001). Chemokines and chemokine receptors also play a crucial role in cancer, especially during metastasis (Zlotnik, 2006). The immune cytolytic activity, along with the existence of complicated associations among various tumour-infiltrated immune cells, elicits immune suppression in kidney cancer (Roufas et al., 2018). Inflammation has been demonstrated to promote the proliferation and metastasis of cancer (Coussens and Werb, 2002). Aran et al. demonstrated that parainflammation, a low-grade form of inflammation, which is widely prevalent in human cancer, is associated with a poor prognosis and p53 mutations (Aran et al., 2016). Co-stimulation and co-inhibition of T-cell activation involves a range of functions that determines T-cell-mediated immune responses (Chen and Flies, 2013). T-cell co-stimulation promotes T-cell activation whereas co-inhibition suppresses T-cell activation (Bour-Jordan et al., 2011). Co-stimulation may be balanced by co-inhibition in the high-risk group (Saleh et al., 1990). Most key immune checkpoints such as PDCD1, CTLA-4, and CD28 were upregulated in the high-risk group. These results above highlight that the high-risk group indicates an immunosuppressive microenvironment. Previous studies also propose a “glutamine steal” scenario, in which cancer cells deprive tumour-infiltrating lymphocytes of the required glutamine, thereby impairing the antitumour immune response (Edwards et al., 2021). The tumour cells may impair the metabolism of immune cells in the tumour microenvironment through high uptake of amino acids, contributing to the immune escape. Thus, the AAM-related gene signatures might down-regulate antitumour immune cells, which enhances the immune escape of ccRCC.

N6-methyladenosine (m^6^A) is the most abundant type of RNA modification in most eukaryotes (Liu and Pan, 2016). Some studies demonstrated that m^6^A modification is closely related to the tumour immune landscape in ccRCC (Zhong et al., 2021). m^6^A RNA methylation participate in the metabolic recombination of tumour cells (An and Duan, 2022). m^6^A is closely linked to various tumours as one of the drivers of tumourigenesis and progression (Yu et al., 2021). Therefore, the special relationship between the expression level of the m^6^A-related gene and the prognostic signature was considered. Previous studies suggested that the risk signature based on m^6^A RNA methylation regulators, METTL3 and METTL14, was of great value for prognosis prediction and closely associated with clinicopathological traits in ccRCC (Zhang et al., 2020). Jiaxun Zhao et al. showed superior survival in patients with either high FTO mRNA or low METTL3 mRNA via survival analysis (Zhao and Lu, 2021). Other studies also demonstrated that the expression levels of METTL3 was intimately associated with tumour size and histological grade (Li et al., 2017). METTL3 can affect cell function and serve as a novel marker for the progression and survival of renal cell carcinoma (Li et al., 2017). Our findings support this correlation, indicating that AAM-related gene signatures could be highly correlated with m^6^A modification and further explain the effect of AAM on tumourigenesis and progression in ccRCC.

There are several limitations to this study. The signature failed to be validated via our cohort in this study, due to the small samples, and further clinical trials are required for validation. Secondly, the molecular mechanisms of several prognostic genes and their relationship with tumour immunity and m^6^A modifications should be further investigated.

## Conclusions

The study developed a novel AAM-related gene signature and constructed a nomogram based on signature and other clinical traits to predict the OS of ccRCC via multiple algorithms. The nomogram can reliably predict the prognosis, which may contribute to optimising precision treatment and further improve the clinical outcome of ccRCC. More importantly, the gene signature was closely related to clinicopathological traits, immune cell infiltration, immune checkpoints, immune-related functions, m^6^A modification, and sensitivity to chemotherapeutic drugs.

## Data Availability

The original contributions presented in the study are included in the article/Supplementary Material, further inquiries can be directed to the corresponding author.

## Supplementary material

Supplementary Figure S1 Landscape of gene mutation in ccRCC.

Supplementary Figure S2 Tumour mutational burden and microsatellite instability analysis; TMB: tumour mutational burden; MSI: microsatellite instability.

Supplementary Figure S3 Validation of the gene expression levels of IYD, ACADSB, and PSAT1 based on renal cancer cell lines via RT-PCR, ns: *p* ≥ 0.05; *: *p* < 0.05; **: *p* < 0.01; ***: *p* < 0.001.
