# Supplementary figures and images for: Novel amino acid metabolism‐related gene signature to predict prognosis in clear cell renal cell carcinoma
Source: Front Genet. 2022 Sep 2;13:982162. doi: 10.3389/fgene.2022.982162 (PMC9478740; doi:10.3389/fgene.2022.982162)

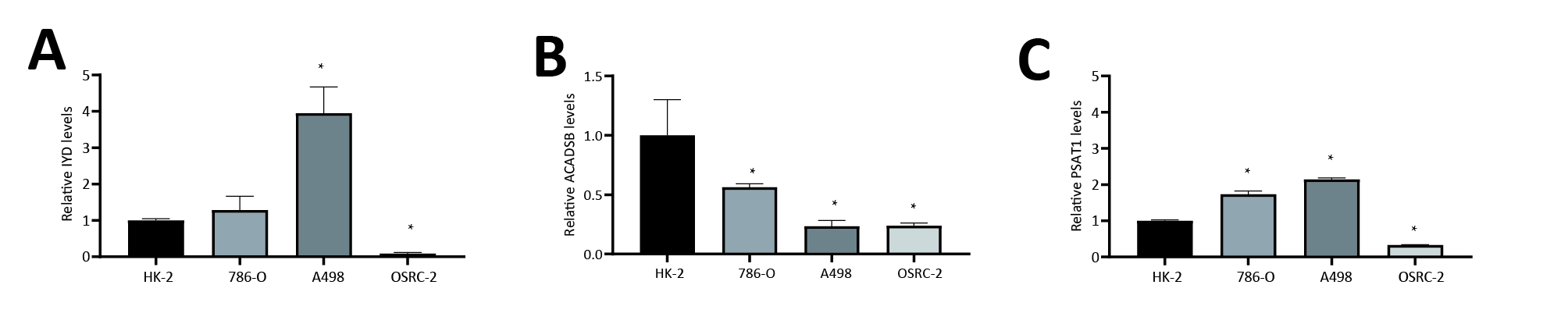

Supplement: Supplementary file 3 [file Image3.tif]

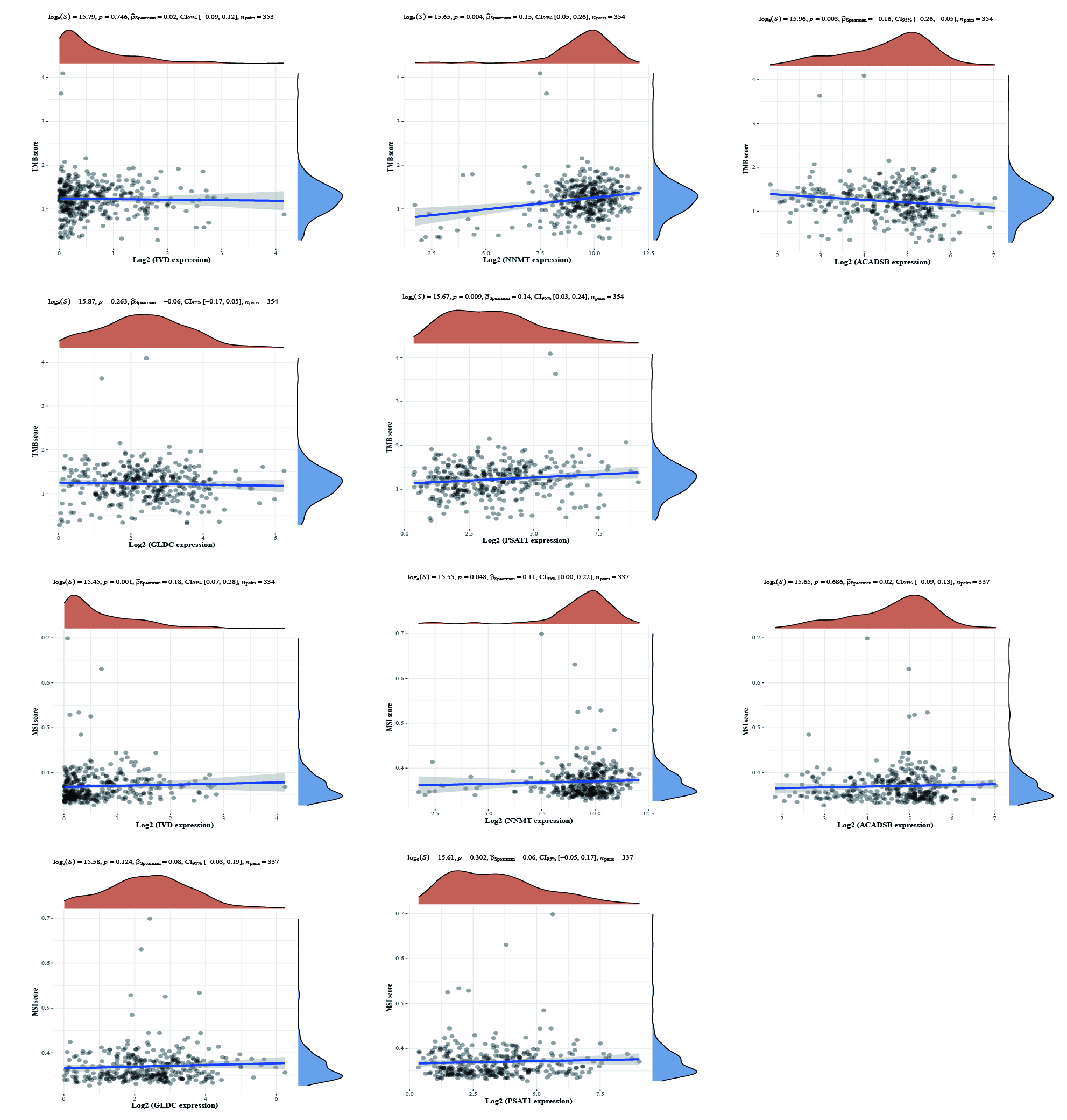

Supplement: Supplementary file 4 [file Image2.TIF]

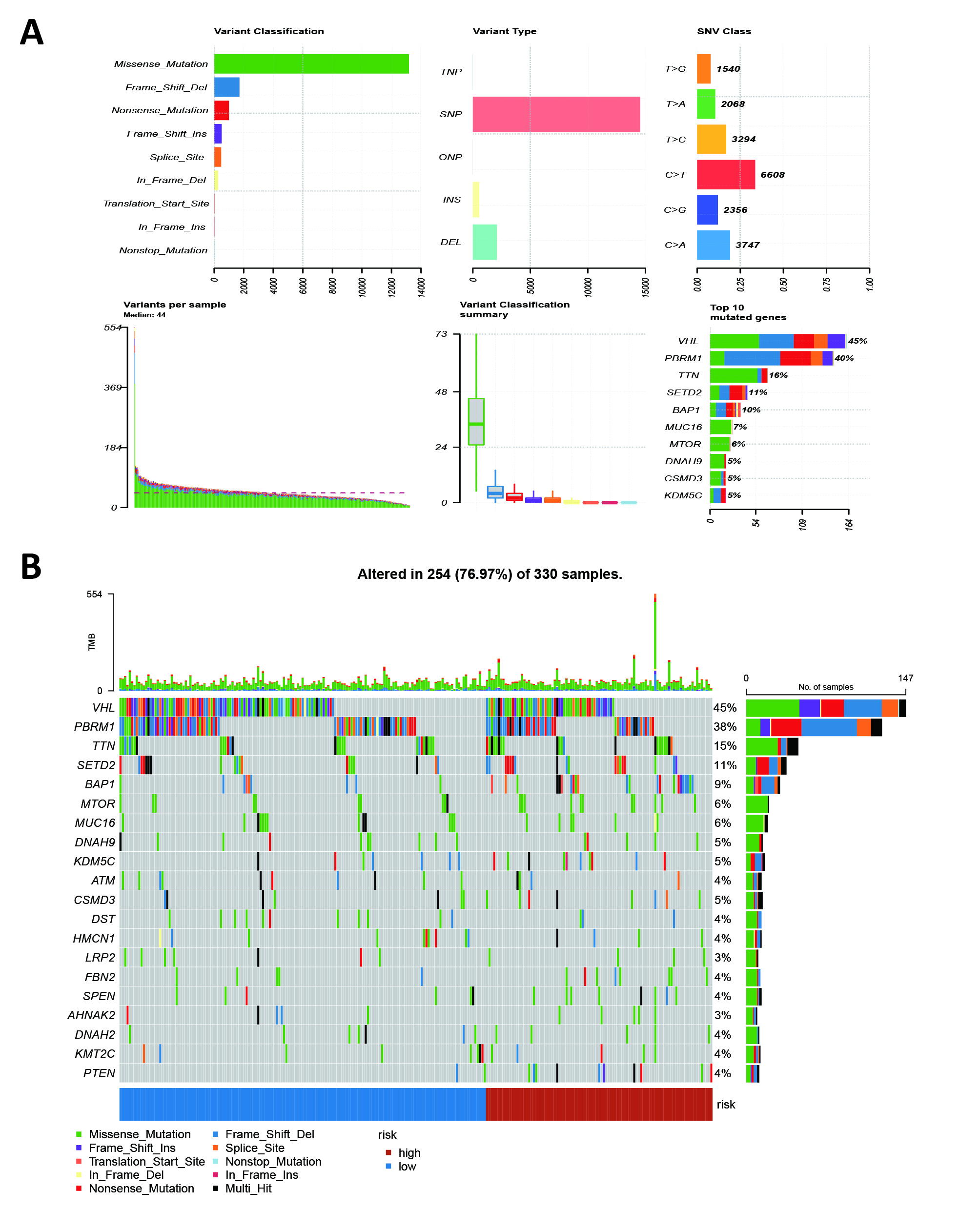

Supplement: Supplementary file 5 [file Image1.TIF]
